# Supplementary material for: tascCODA: Bayesian Tree-Aggregated Analysis of Compositional Amplicon and Single-Cell Data
Source: Front Genet. 2021 Dec 7;12:766405. doi: 10.3389/fgene.2021.766405 (PMC8689185; doi:10.3389/fgene.2021.766405)
Supplement: Supplementary file 1 [file DataSheet1.PDF]

Supplementary Material

1 SUPPLEMENTARY DATA

1.1 Notation overview

This section gives an overview over the inputs and parameters used by tascCODA:

Data inputs

- $Y \in \mathbb{R}^{n \times p}$ is the count matrix of features $j = 1 \ldots p$ in samples $i = 1 \ldots n$. $\bar{Y}_i = \sum_{j=1}^{p} Y_{i,j}$ is the sequencing depth of sample i.
- $X \in \mathbb{R}^{n \times d}$ is the covariate matrix of covariates $l = 1 \ldots d$ for samples $i = 1 \ldots n$.
- $\mathcal{T}$ is a multifurcating tree structure with p leaves and t internal nodes defined by the ancestor matrix $A \in \{0, 1\}^{p \times v}$, with $v = p + t$

Latent parameters

- $\mathbf{a}_i = (a_{1,i}, \ldots a_{p,i}); a_{j,i} \geq 0$ is the probability vector of the Dirichlet-Multinomial distribution for sample i.
- α_j is the base (intercept) parameter for feature j.
- $\beta_{l,j}$ is the effect of covariate l on feature j.
- $\hat{\beta}_{l,k}$ is the effect of covariate l on tree node k.
- $\tilde{\beta}_{0,l,k}$ is the spike portion of the spike-and-slab LASSO prior for covariate l and tree node k with parameters $\sigma_{0,l,k}$ and $b_{0,l,k}$.
- $\tilde{\beta}_{1,l,k}$ is the slab portion of the spike-and-slab LASSO prior for covariate l and tree node $k$with parameters $\sigma_{1,l,k}$ and $b_{1,l,k}$.
- θ is the mixture coefficient of the spike-and-slab LASSO prior.

Tuning parameters/hyperparameters

- λ_0 is the shrinkage parameter for the spike portion, default $\lambda_0 = 50$.
- $\lambda_{1,k}$ is the node-specific shrinkage parameter for the slab portion of the prior on node k, with mean value λ_1, default $\lambda_1 = 5$.
- ϕ is the aggregation bias parameter for scaling the slab shrinkage $\lambda_{1,k}$

1.2 Hyperparameters for the spike-and-slab LASSO prior

We want to shed some additional light on the role of the hyperparameters $\lambda_0, \lambda_1, \theta$ in the spike-and-slab LASSO prior (Ročková and George (2018)). For simplicity and because the model is symmetric with respect to the covariates, we assume $d = 1$ and thus refrain from indexing parameters with the covariate. For one node $\hat{\beta}_k$, the prior is a mixture of two double-exponential distributions $\psi_0(\hat{\beta}_k)$ and $\psi_1(\hat{\beta}_k)$ (Figure S1A) whose share is determined by θ:

$$p(\hat{\beta}_k|\theta) = \theta\psi_1(\hat{\beta}_k) + (1-\theta)\psi_0(\hat{\beta}_k) \tag{S1}$$

$$\psi_1(\hat{\beta}_k) = \frac{\lambda_1}{2}e^{-\lambda_1|\hat{\beta}_k|} \tag{S2}$$

$$\psi_0(\hat{\beta}_k) = \frac{\lambda_0}{2}e^{-\lambda_0|\hat{\beta}_k|} \tag{S3}$$

The double-exponential density (S2) has a peak at zero for large values of λ, which decreases with λ (Bai et al. (2020)). Thus, setting $\lambda_0 \gg \lambda_1$ in the mixture density (S1) results in a product of a peaked "spike" (ψ_0) and a diffuse "slab" (ψ_1) component (Figure S1B). Interestingly, Ročková and George (2018) showed that the spike-and-slab LASSO prior can be reformulated as a penalized likelihood method that is, for fixed θ:

$$pen(\hat{\beta}_k|\theta) = -\lambda_1|\hat{\beta}_k| + \log(\frac{p^*_\theta(0)}{p^*_\theta(\hat{\beta}_k)}) \tag{S4}$$

where

$$p^*_\theta(b) = \frac{\theta\frac{\lambda_1}{2}e^{-\lambda_1|b|}}{\theta\frac{\lambda_1}{2}e^{-\lambda_1|b|} + (1-\theta)\frac{\lambda_0}{2}e^{-\lambda_0|b|}} \tag{S5}$$

In the case of $\lambda_0 = \lambda_1$, the log-term in (S4) vanishes, and the penalty is equivalent to the standard LASSO (Tibshirani (1996)).

After making the weight θ data-adaptive by a Beta prior (Equation (9)), we turn our attention to the double-exponential parameters. We show the influence of each parameter on the solution by simulations on one of the randomly generated datasets from the simulation study with $p = 10$ features. From Figure S2, we can see that the ground truth assumption are effects on nodes 0, 4, and 12, with the latter node affecting features 7 and 8. We first fix $\lambda_1 = 1$, and vary λ_0 on a scale between 1 and 1000. Figure S1C shows that the effects $\hat{\beta}$ quickly stabilize with the three true effects being clearly separated from all other effects, which are close to zero. This stabilization was also explained by Ročková and George (2018) and is rooted in the fact that larger values of λ_0 only narrow the spike, which does not affect the solution after some point. We can thus simply set λ_0 to a relatively large value, the default in tascCODA is $\lambda_0 = 50$. When $\lambda_0 = \lambda_1$, we can see the typical parameter curve of a LASSO model, where the true effects are the last to approach zero (Figure S1D).

Because $\lambda_1 \to \lambda_0$ approaches the $\mathcal{L}_1$ penalty of the LASSO, which will eventually force all effects towards zero, leaving $\lambda_0 = 50$ and increasing λ_1 shows a similar behavior (Figure S1E). Only the true effects are significantly larger than zero once λ_1 reaches a value of approximately 0.1. After a certain point ($\lambda_1 \approx 10$), the penalty becomes so large that all effects vanish. We utilize the regularizing behavior by scaling λ_1 depending on the number of leaves that a node influences to put a preference on nodes on different levels of the tree (Equation 10). The direction and steepness of the preference is expressed by the parameter ϕ, with $\phi = 0$ giving equal treatment to all nodes. The default overall size of the penalty, $\lambda_1 = 5$, is chosen in a way that the parameters $\lambda_{1,k} \in (0, 10]$ stay in the range of values that were recommended by Ročková and George (2018) for all k. Figure S1F shows how the results change with different values of ϕ.

For $\phi <= 0$, favoring high-level aggregations, the model selects the three ground truth nodes. When $\phi > 1$, tips are penalized considerably less than internal nodes and the effect on node 12 is replaced by equal-sized effects on its children, nodes 7 and 8. Also, for $\phi < 0$, effects on nodes that are high in the tree (large k) are different from zero, but smaller than the significance threshold (dashed line), while for $\phi > 0$, this is the case for leaf nodes.

1.3 Experimental data preprocessing

1.3.1 Single-cell RNA-seq analysis of ulcerative colitis in humans

We obtained the data on ulcerative colitis from from Single Cell Portal (accession ID SCP259) and the analysis code from github. In total, the data consists of 365,492 transcriptomes from 12 healthy donors and 18 donors with UC providing non-inflamed and inflamed tissue samples. We used the 51 different cell types found in the original analysis, but considered every replicate as an independent sample, as done in a re-analysis by Büttner et al. (2020) on the same dataset. Biopsies from two different tissue regions, the Epithelium ('Epi' - 24 healthy, 21 non-inflamed, 16 inflamed samples) and the underlying Lamina Propria ('LP' - 24 healthy, non-inflamed, and inflamed samples each), were divided by enzymatic digestion. We inferred the cell lineage tree from the Methods section of Smillie et al. (2019) (Figure S11).

1.3.2 Analysis of the human gut microbiome under Irritable Bowel Syndrome

The raw 16S rRNA sequences (avilable at the Short Read Archive, accession number PRJNA373876) were re-processed using DADA2, version 1.21.0 (Callahan et al. (2016)). After primer and quality filtering (minimum read length: 150bp, maximum errors per read: 3, reads trimmed at first base with quality below: 10), inference of ASVs and removal of chimeras, the taxonomy of the inferred ASVs was determined with the Silva database, version 138.1 (Quast et al. (2013); Yilmaz et al. (2014)). Samples with a total read count of less than 500 (n=0) were discarded and ASVs assigned to Eukaryota (n=0) or belonging to an unknown Phylum (n=1) were removed, yielding a final count table with 709 ASVs along with a taxonomic tree.

2 SUPPLEMENTARY TABLES AND FIGURES

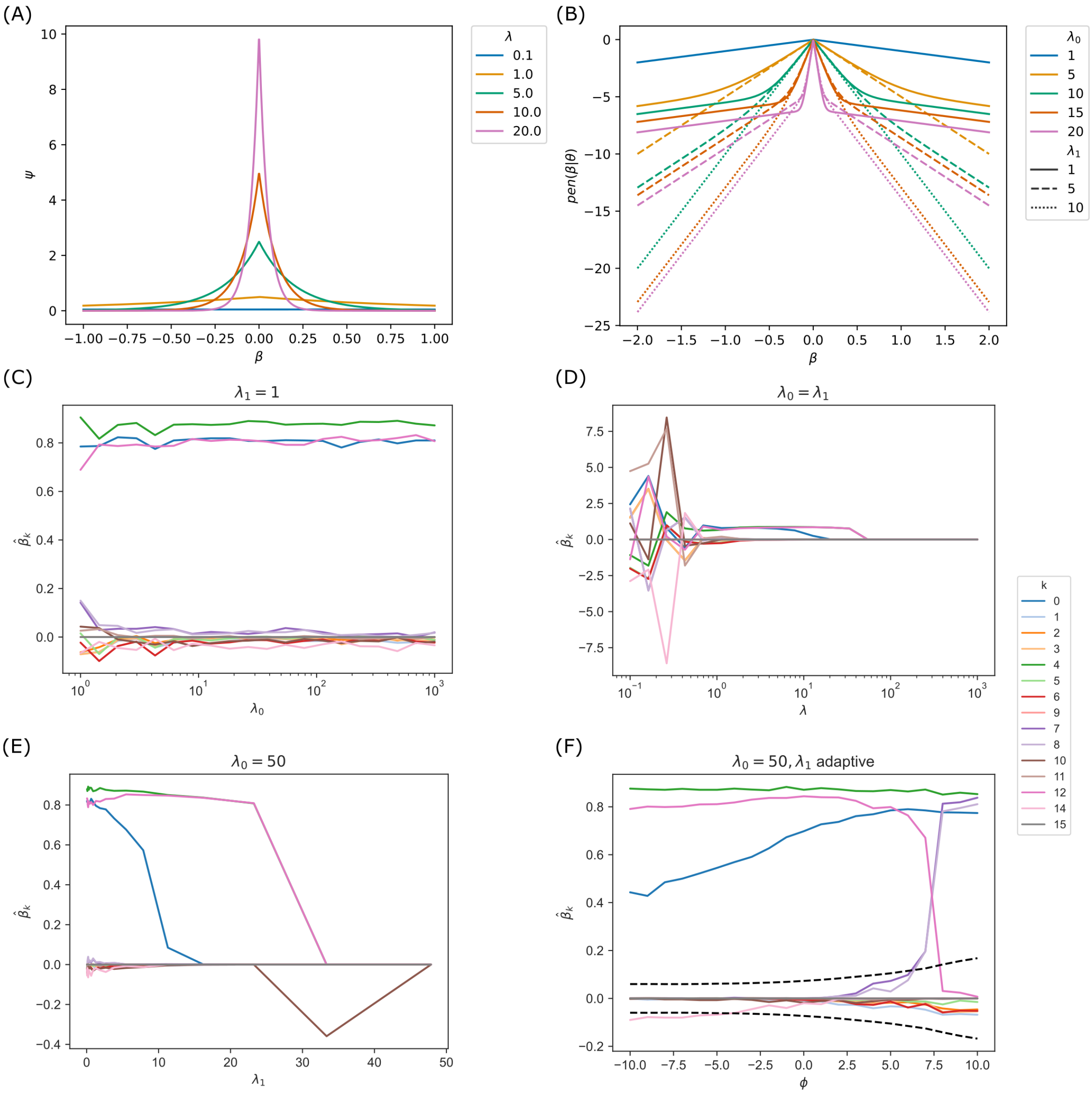

Figure S1. Parameters in the spike-and-slab LASSO penalty. **(A)** The double exponential density $\psi(\beta, \lambda)$ for different values of λ. The density becomes more peaked with increasing λ. **(B)** The likelihood penalty (Equation (S4)) introduced by different parametrizations of the spike-and-slab LASSO prior ($\theta = 0.1$). For larger effect sizes β, the penalty is driven by the slab parameter λ_1 (lines with the same style are close together). For smaller effect sizes β, the penalty is driven by the spike parameter λ_0 (lines with the same color are close together). If $\lambda_0 = \lambda_1$, the penalty is linear and equivalent to the LASSO penalty $\lambda_0 \beta$. **(C-F)** Effect of different parameters on the effects $\hat{\beta}_k$ determined by tascCODA. For all simulations, a realization of the dataset in Supplementary Figure S2 was used. The nodes 13, 16 and 17 are singularities and were thus deleted before model application. **(C)** Solutions found by tascCODA when varying values of λ_0 and constant $\lambda_1 = 1$. The effects $\hat{\beta}_k$ stabilize and increasing λ_0 has no effect. **(D)** Solutions found by tascCODA in a LASSO-equivalent setting when varying values of $\lambda_0 = \lambda_1 = \lambda$. With increasing λ, more effects $\hat{\beta}_k$ go to 0. **(E)** Solutions found by tascCODA when varying values of λ_1 and constant $\lambda_0 = 50$. With increasing λ_1, a similar effect to the LASSO can be seen, where all effects are eventually approaching 0. **(F)** Solutions found by tascCODA when varying the tree level bias ϕ. $\lambda_0 = 50, \lambda_{1,k}$ as in Equation 10. The dashed black lines show the significance threshold (Equation 11).

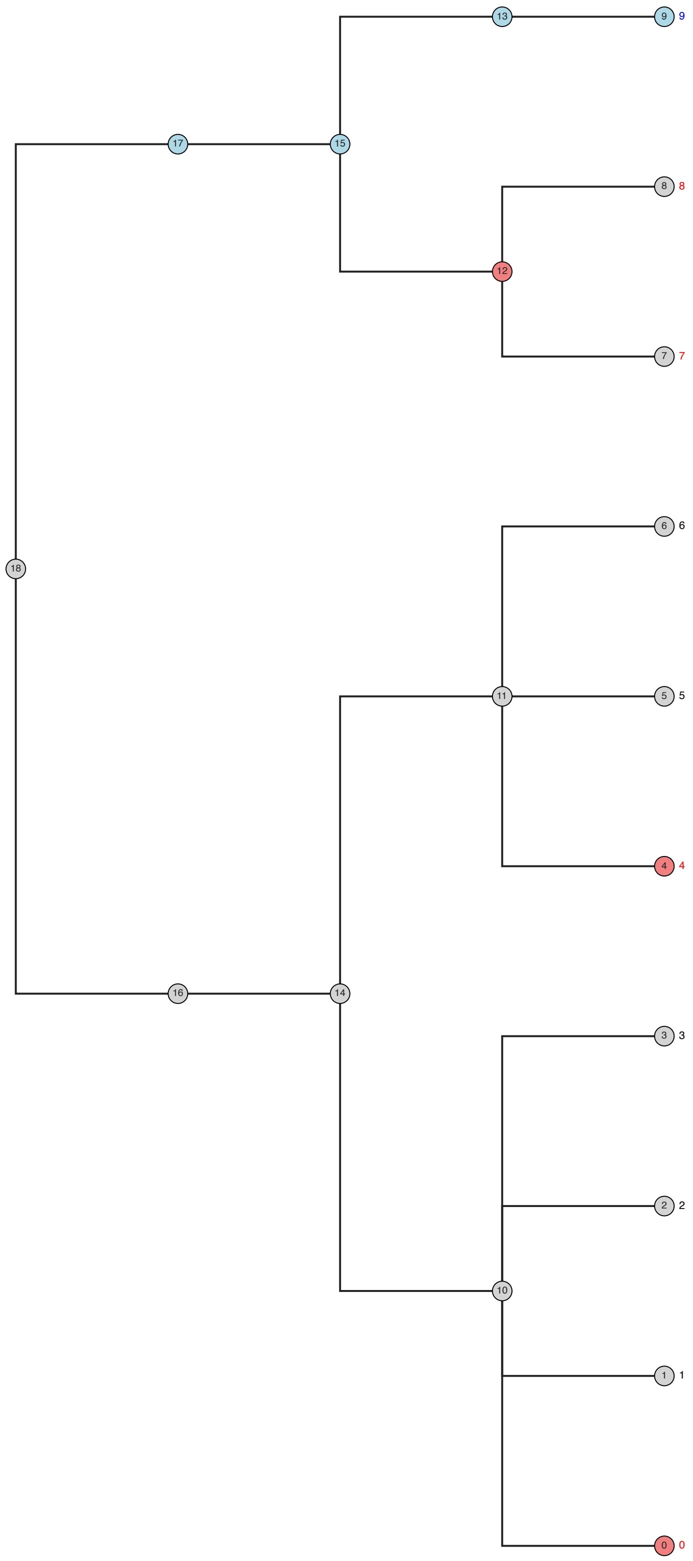

Figure S2. Randomly generated tree structure for synthetic data benchmark, $p = 10$ tips. The red nodes were selected to be affected by the condition, causing the red tips to be differentially abundant. The blue tip is the reference feature, which forces the effects on all blue nodes to be 0.

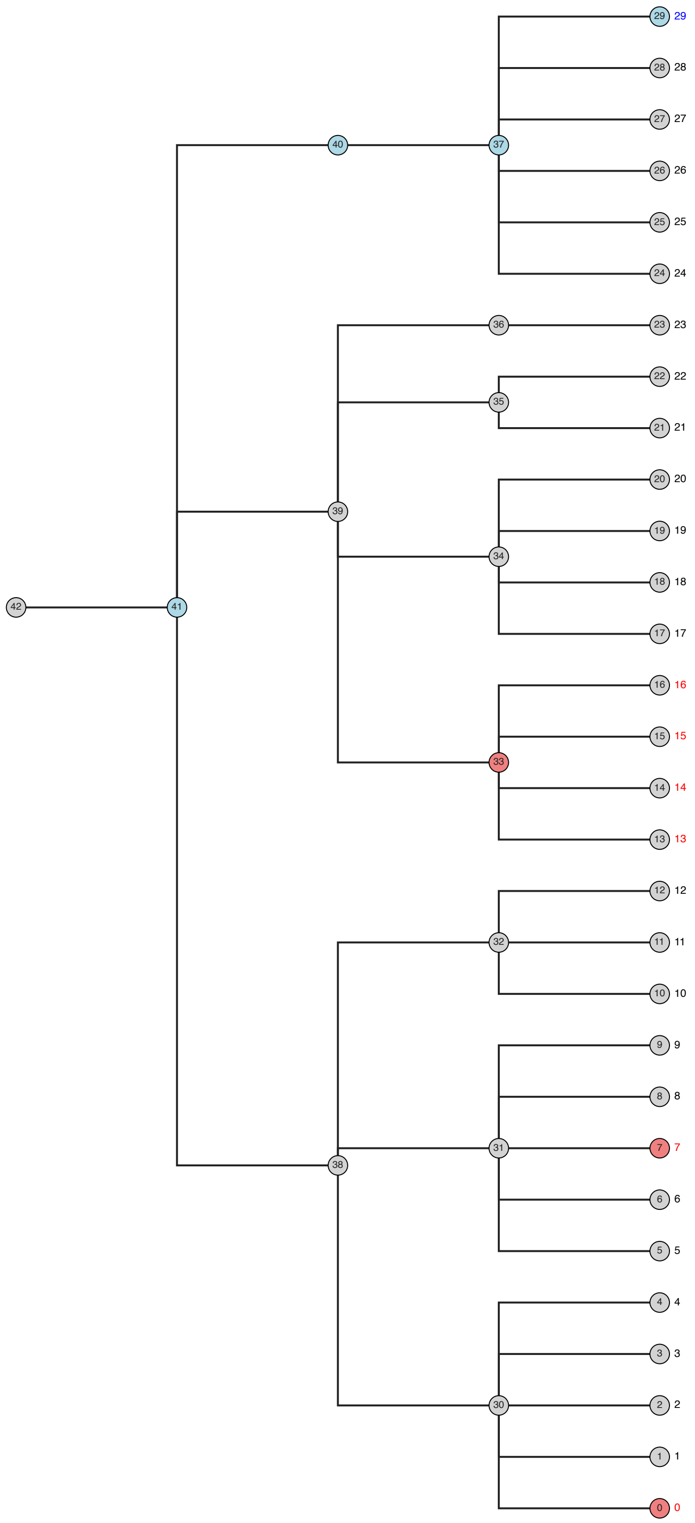

Figure S3. Randomly generated tree structure for synthetic data benchmark, $p = 30$ tips. The red nodes were selected to be affected by the condition, causing the red tips to be differentially abundant. The blue tip is the reference feature, which forces the effects on all blue nodes to be 0.

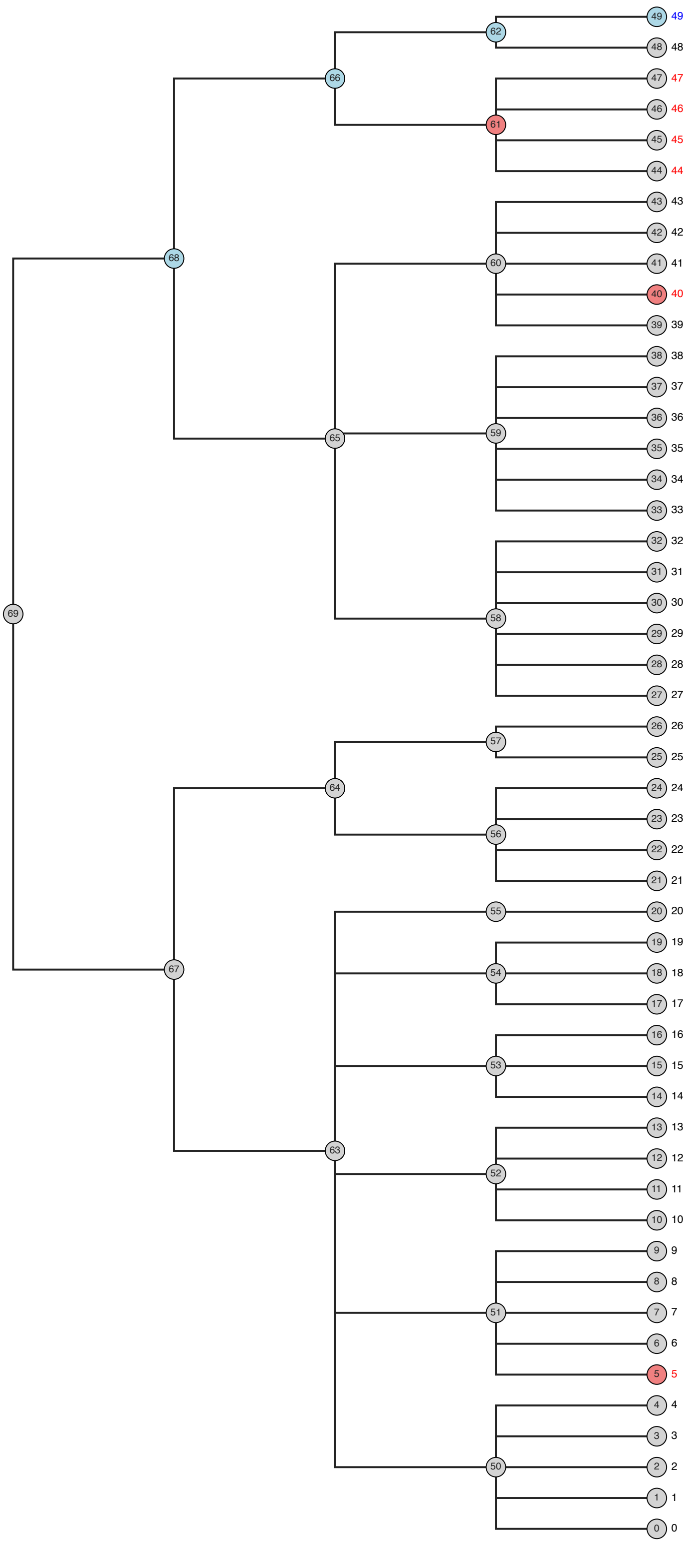

Figure S4. Randomly generated tree structure for synthetic data benchmark, $p = 50$ tips. The red nodes were selected to be affected by the condition, causing the red tips to be differentially abundant. The blue tip is the reference feature, which forces the effects on all blue nodes to be 0.

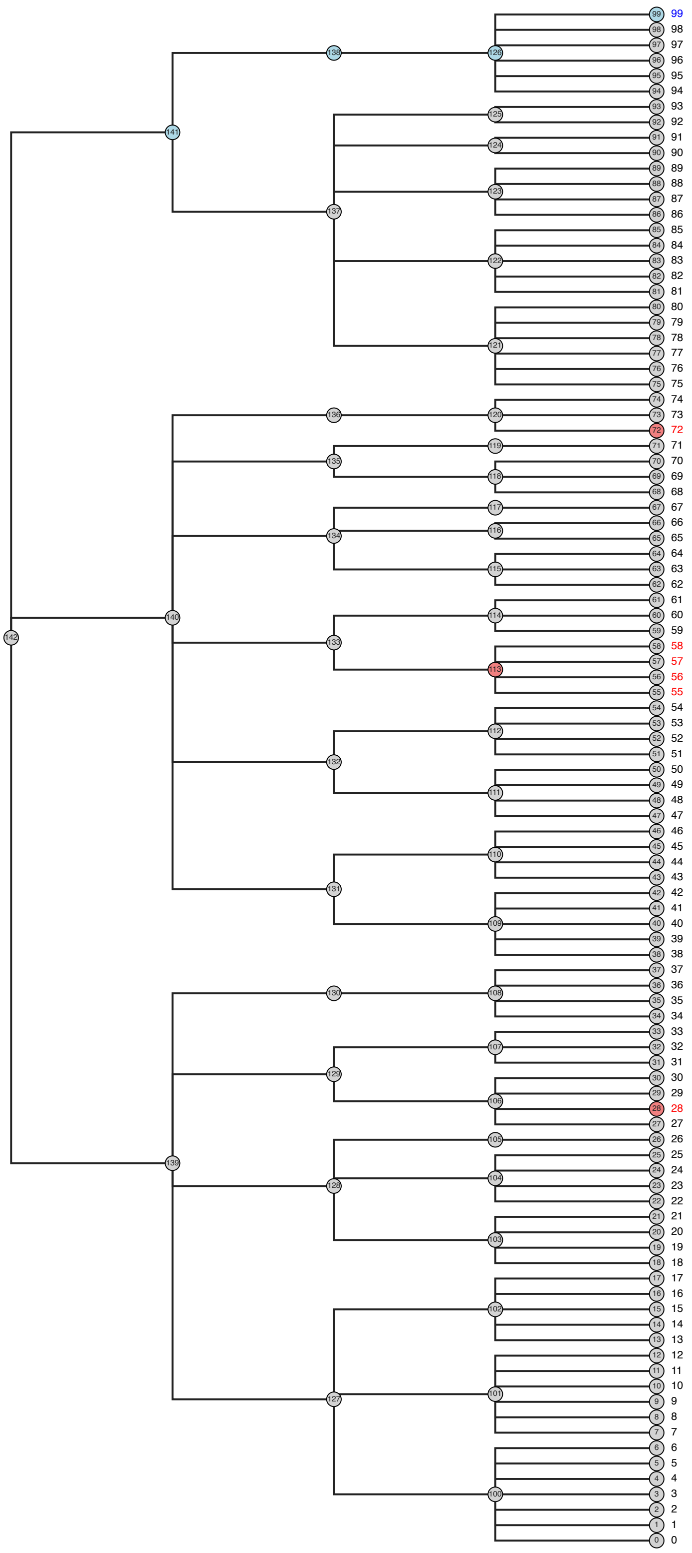

Figure S5. Randomly generated tree structure for synthetic data benchmark, $p = 100$ tips. The red nodes were selected to be affected by the condition, causing the red tips to be differentially abundant. The blue tip is the reference feature, which forces the effects on all blue nodes to be 0.

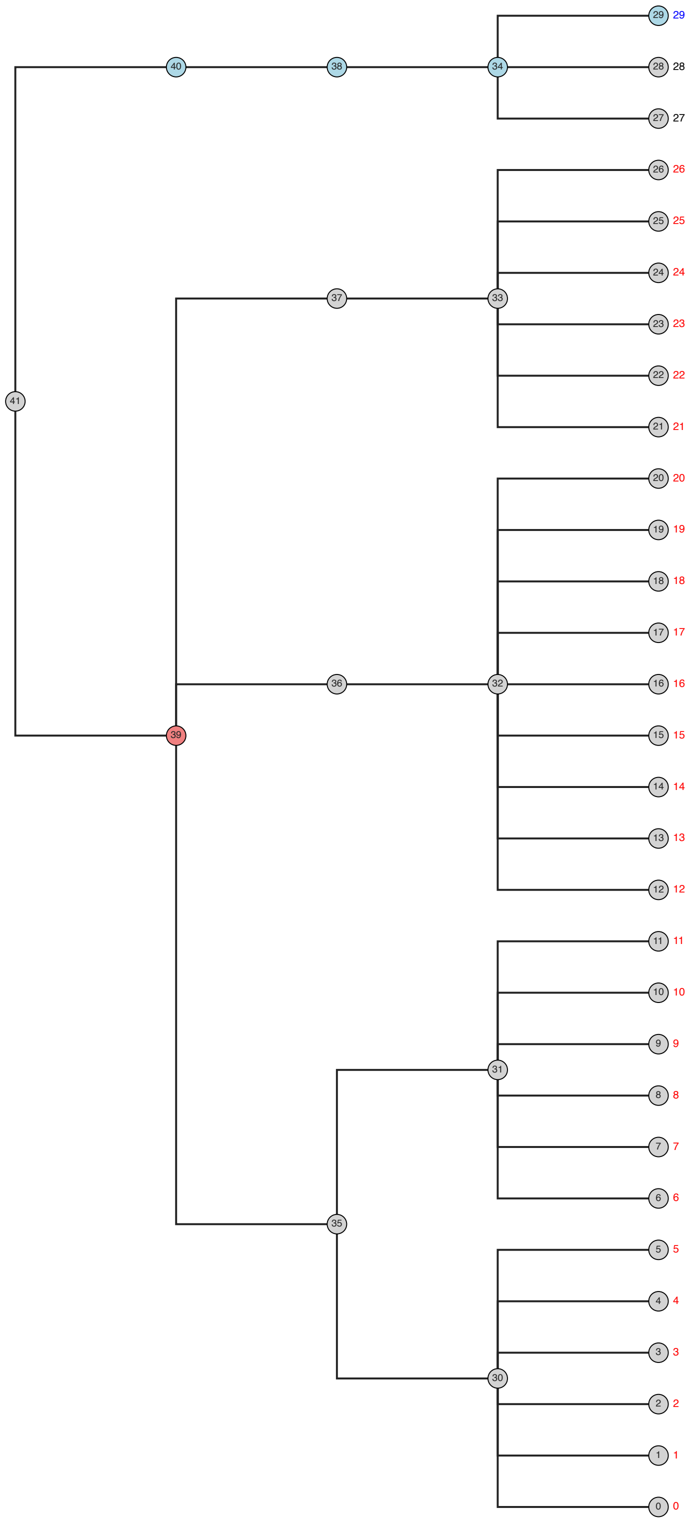

Figure S6. Randomly generated tree structure for synthetic data benchmark with one effect near the root of the tree, $p = 30$ tips. The red nodes were selected to be affected by the condition, causing the red tips to be differentially abundant. The blue tip is the reference feature, which forces the effects on all blue nodes to be 0.

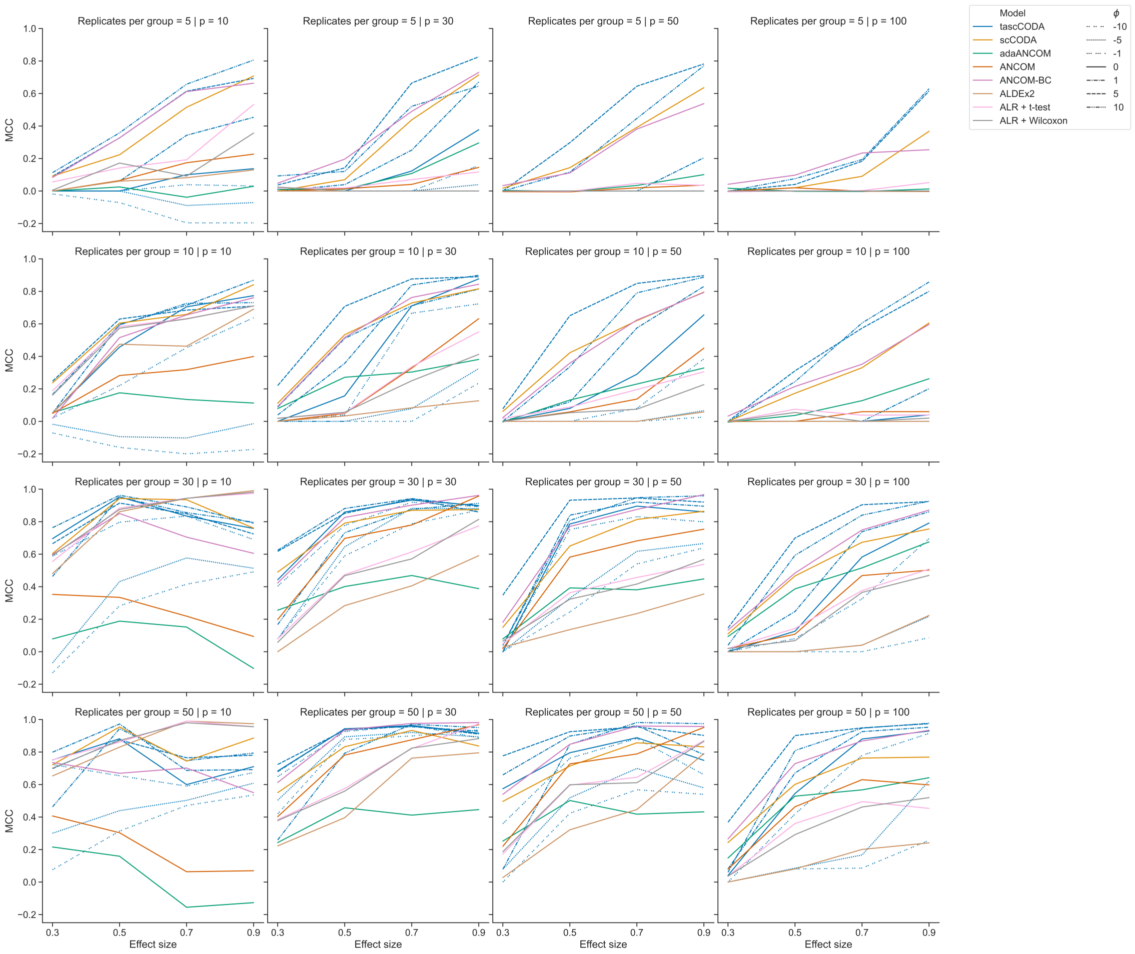

Figure S7. Matthews correlation coefficient (MCC) of tascCODA and other methods on simulated data with one binary covariate (differential abundance testing). Plots are grouped by the number of simulated components p, the number of samples per group and the effect size β. For tascCODA, different values of ϕ were tested (dashed blue lines).

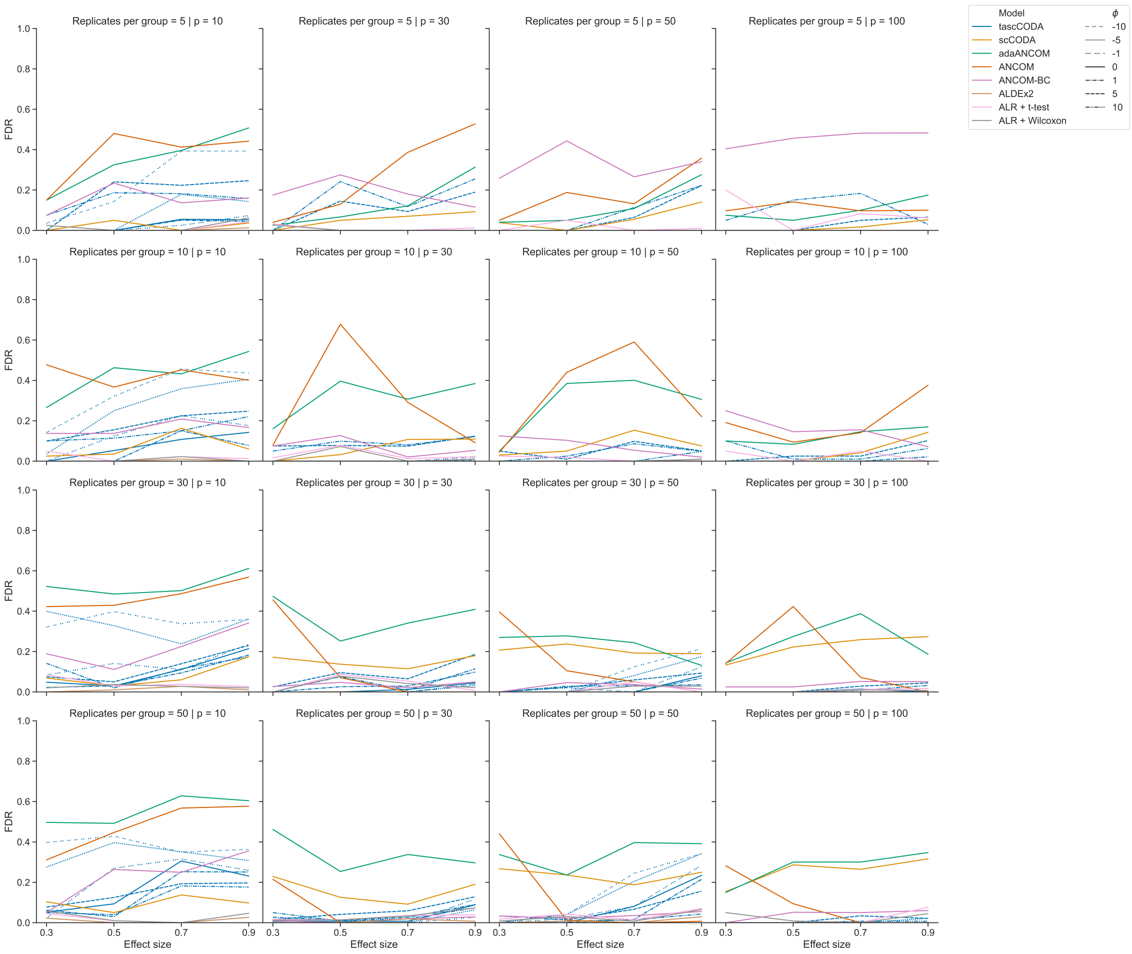

Figure S8. False discovery rate (FDR) of tascCODA and other methods on simulated data with one binary covariate (differential abundance testing). Plots are grouped by the number of simulated components p, the number of samples per group and the effect size β. For tascCODA, different values of ϕ were tested (dashed blue lines).

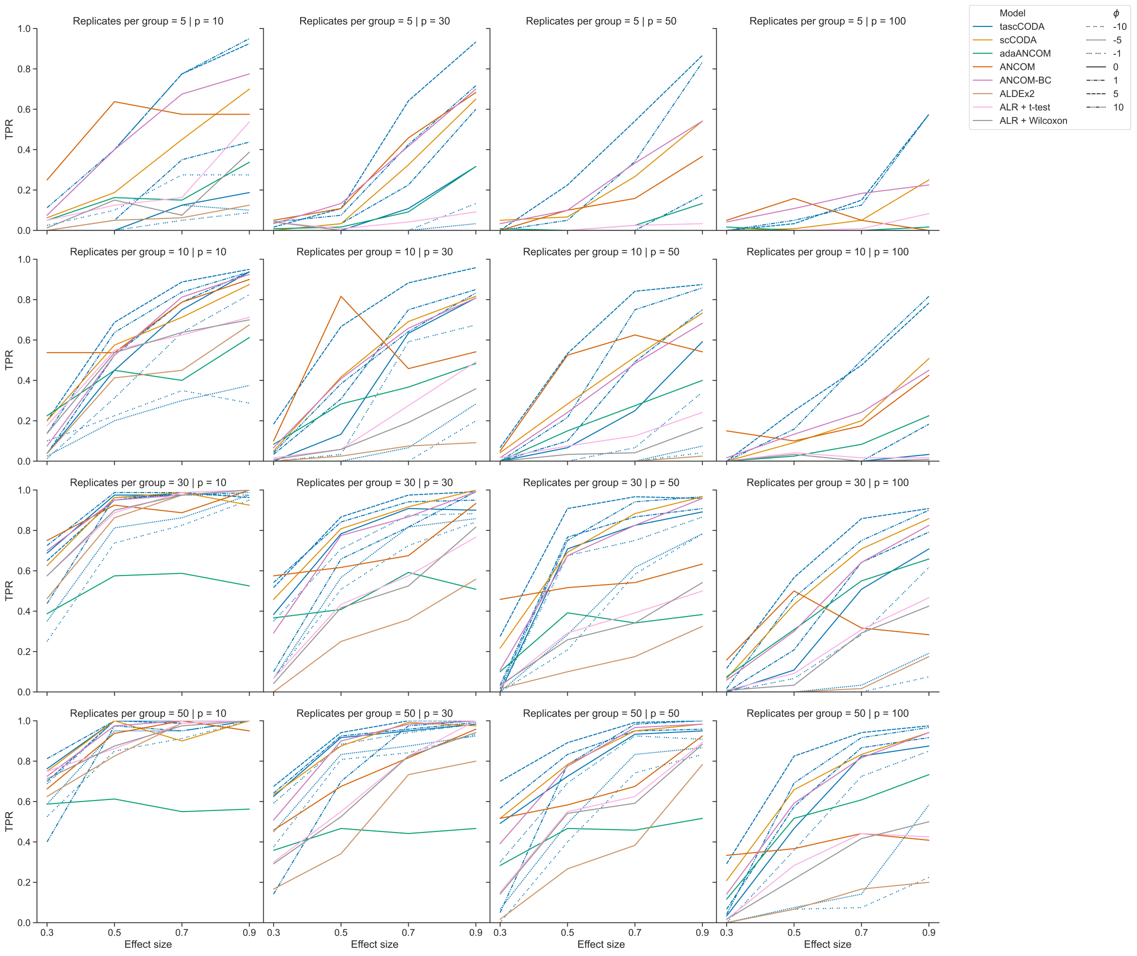

Figure S9. True positive rate (TPR) of tascCODA and other methods on simulated data with one binary covariate (differential abundance testing). Plots are grouped by the number of simulated components p, the number of samples per group and the effect size β. For tascCODA, different values of ϕ were tested (dashed blue lines).

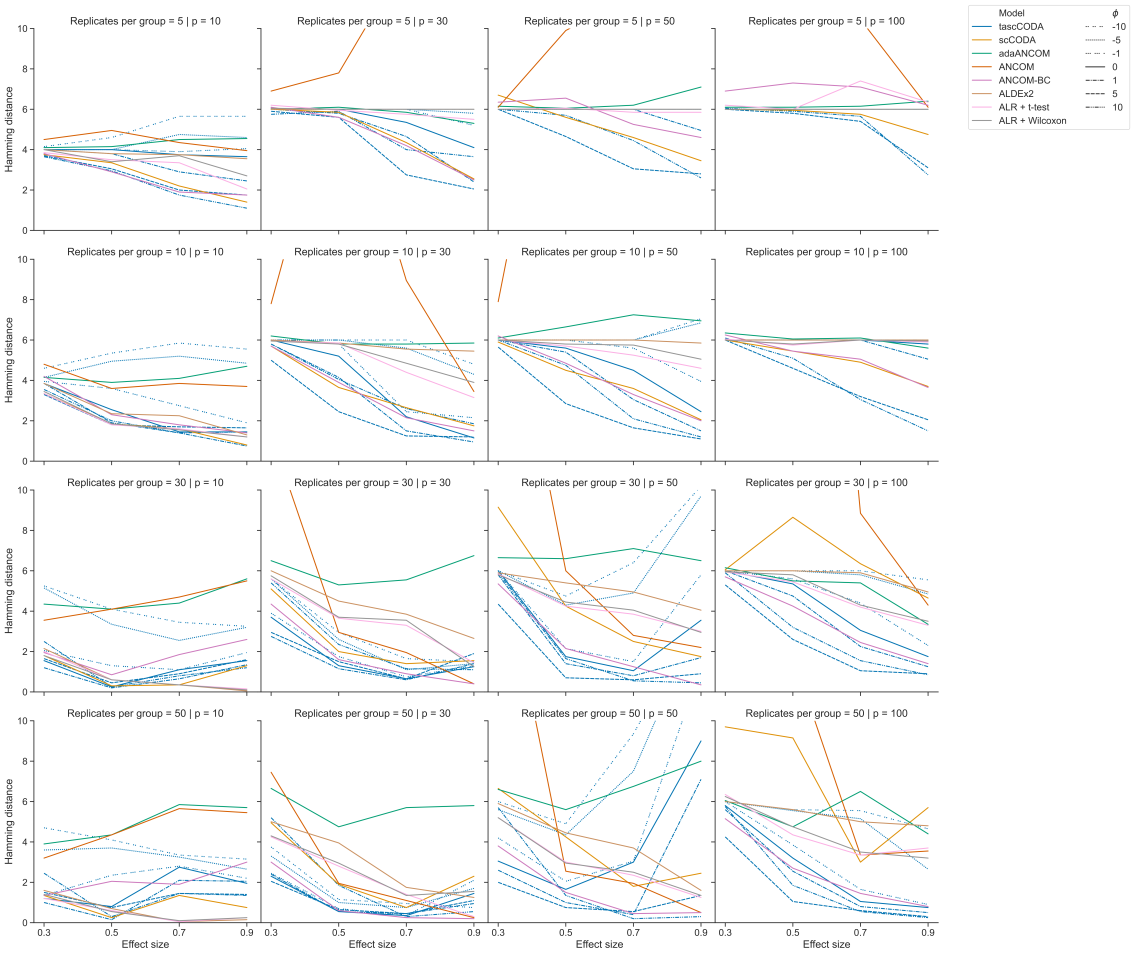

Figure S10. Hamming distance between ground truth and affected features determined by tascCODA and other methods on simulated data with one binary covariate (differential abundance testing). Plots are grouped by the number of simulated components p, the number of samples per group and the effect size β. For tascCODA, different values of ϕ were tested (dashed blue lines).

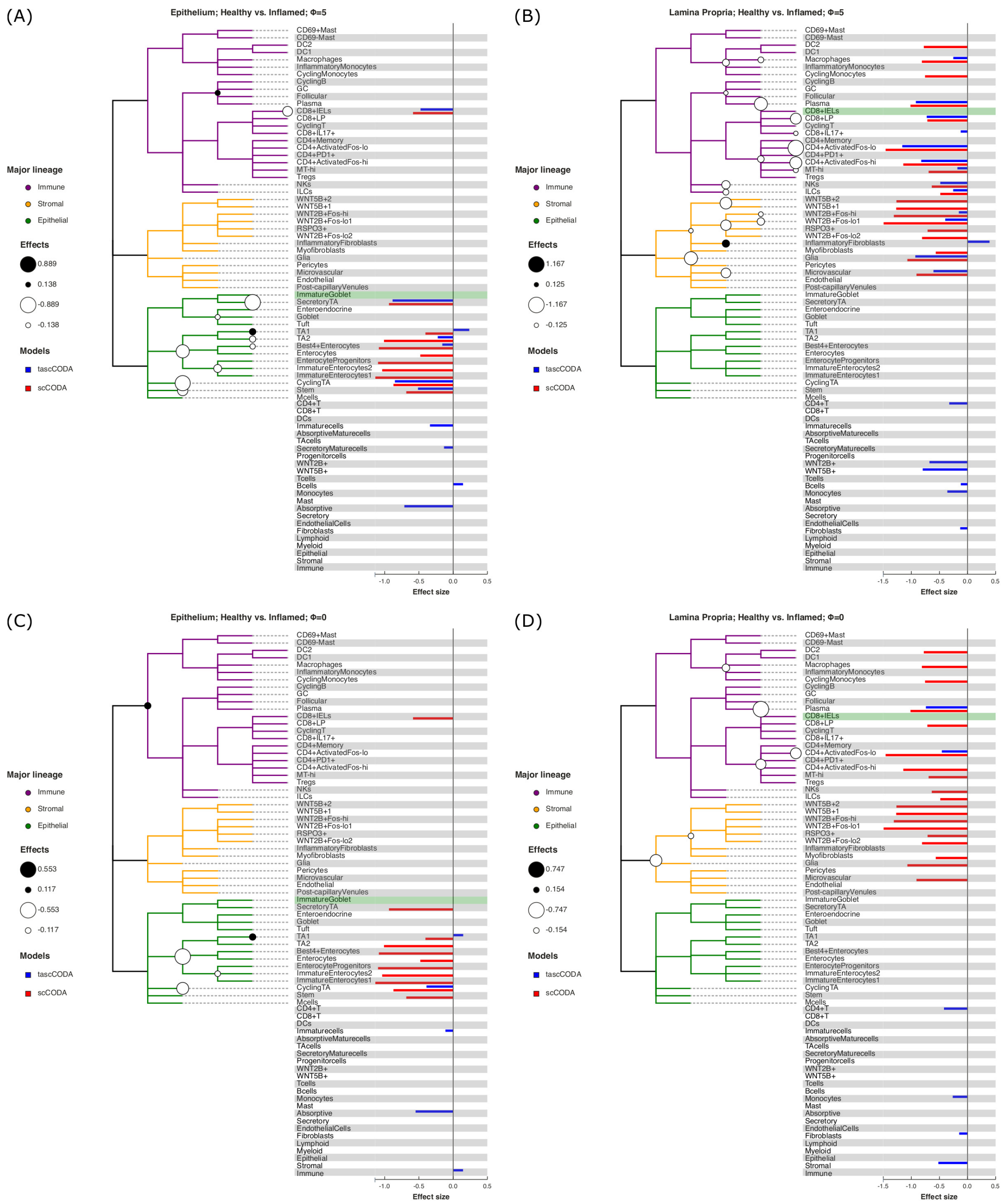

Figure S11. Behavior of tascCODA on scRNA-seq data. All plots show the case of comparing healthy control samples to inflamed tissue samples of UC patients in the data of Smillie et al. (2019). White and black circles on the cell lineage tree show the effects found by tascCODA, which are also shown as blue bars on the right side of each plot. The bars below the tree depict effects on internal nodes, with lower positions in the diagram corresponding to nodes closer to the root. For comparison, the red bars indicate effects found by scCODA, which only operates on the tips of the tree, on the same data. The green-shaded area shows the reference cell type that was used for both models. **(A)** $\phi = 5$, Epithelium. **(B)** $\phi = 5$, Lamina Propria. **(C)** $\phi = -3$, Epithelium. **(D)** $\phi = -3$, Lamina Propria.

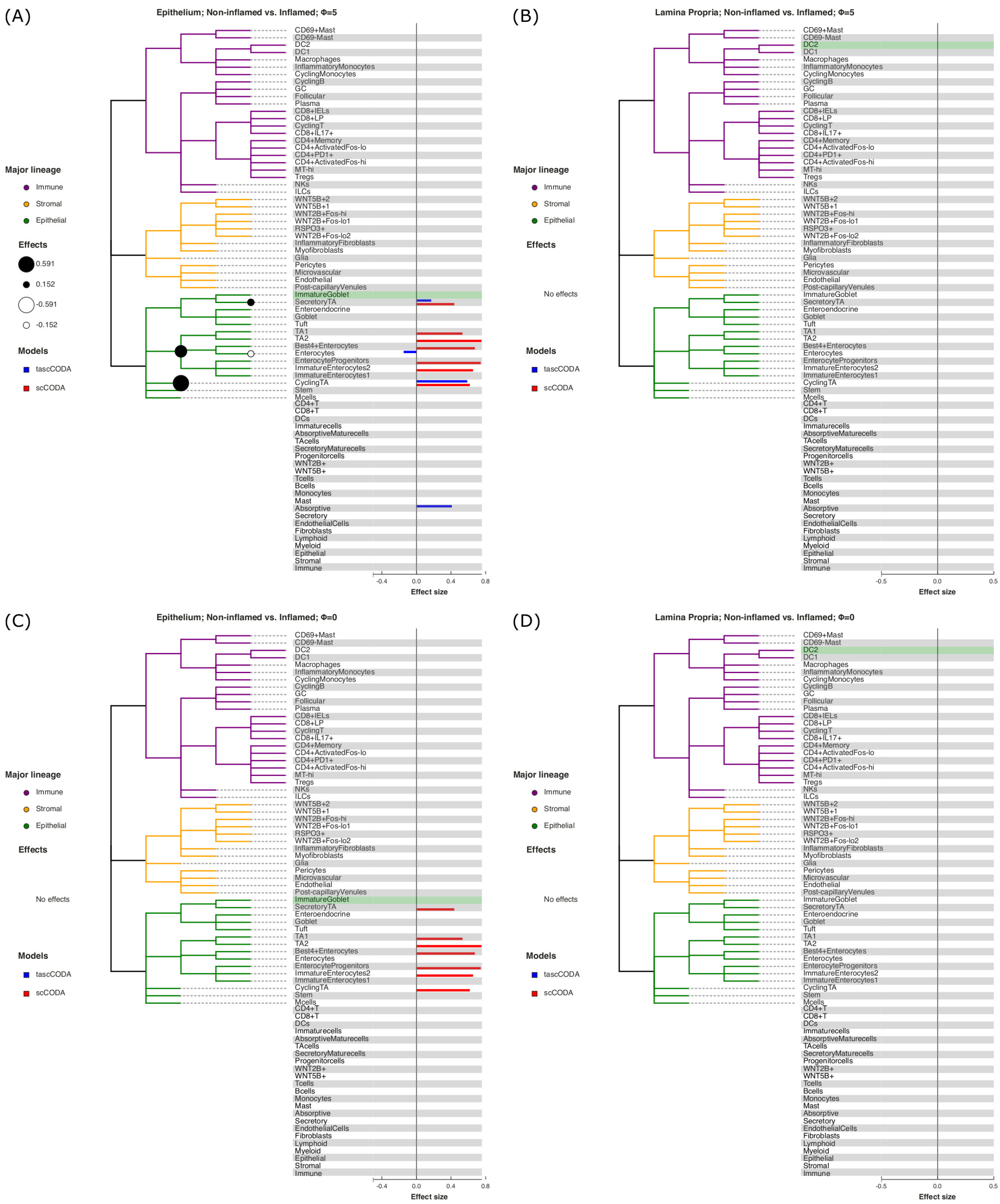

Figure S12. Behavior of tascCODA on scRNA-seq data. All plots show the case of non-inflamed to inflamed tissue samples of UC patients in the data of Smillie et al. (2019). White and black circles on the cell lineage tree show the effects found by tascCODA, which are also shown as blue bars on the right side of each plot. The bars below the tree depict effects on internal nodes, with lower positions in the diagram corresponding to nodes closer to the root. For comparison, the red bars indicate effects found by scCODA, which only operates on the tips of the tree, on the same data. The green-shaded area shows the reference cell type that was used for both models. **(A)** $\phi = 5$, Epithelium. **(B)** $\phi = 5$, Lamina Propria. **(C)** $\phi = -3$, Epithelium. **(D)** $\phi = -3$, Lamina Propria.

Table S1. Credible effects, highest density intervals, standard deviations and credibility threshold δ determined by tascCODA on epithelial biopsies from Smillie et al. (2019), $\phi = 5$. Abbreviations for scenarios: Healthy (H), Non-inflamed (N), and Inflamed (I).

Scenario	Node	Effect	HDI 3%	HDI 97%	SD	δ
Epithelium - H vs. N	ImmatureEnterocytes1	-0.647	-0.984	-0.309	0.181	0.148
	Enterocytes	-0.211	-0.637	0.054	0.211	0.148
	TA1	0.280	-0.022	0.475	0.134	0.148
Epithelium - H vs. I	Stem	-0.518	-1.000	0.004	0.286	0.135
	CyclingTA	-0.855	-1.144	-0.592	0.146	0.135
	Best4+Enterocytes	-0.163	-0.893	0.141	0.303	0.135
	TA2	-0.229	-0.802	0.129	0.282	0.135
	TA1	0.240	-0.159	0.730	0.265	0.135
	SecretoryTA	-0.889	-1.334	-0.477	0.228	0.135
	CD8+IELs	-0.481	-1.024	0.018	0.303	0.135
	Immaturecells	-0.343	-0.951	0.091	0.317	0.132
	SecretoryMaturecells	-0.138	-0.526	0.082	0.177	0.132
	Bcells	0.149	-0.068	0.525	0.178	0.130
	Absorptive	-0.717	-1.209	-0.110	0.299	0.126
Epithelium - N vs. I	CyclingTA	0.591	0.302	0.907	0.161	0.144
	Enterocytes	-0.152	-0.856	0.121	0.294	0.144
	SecretoryTA	0.174	-0.075	0.677	0.227	0.144
	Absorptive	0.413	-0.031	0.742	0.247	0.135

Table S2. Credible effects, highest density intervals, standard deviations and credibility threshold δ determined by tascCODA on Lamina Propria biopsies from Smillie et al. (2019), $\phi = 5$. Abbreviations for scenarios: Healthy (H), Non-inflamed (N), and Inflamed (I). For the N vs. I scenario, no credible effects were found.

Scenario	Node	Final Parameter	HDI 3%	HDI 97%	SD	Delta
LP - H vs. N	ImmatureGoblet	0.154	-0.161	0.787	0.270	0.131
	Microvascular	-0.354	-0.899	0.089	0.292	0.131
	Glia	-0.351	-0.867	0.083	0.278	0.131
	ILCs	-0.189	-0.710	0.125	0.242	0.131
	CD4+ActivatedFos-hi	-0.144	-0.544	0.100	0.184	0.131
	CD4+ActivatedFos-lo	-0.608	-1.048	-0.162	0.233	0.131
	CD8+LP	-0.169	-0.655	0.105	0.220	0.131
	Plasma	-0.472	-0.895	0.006	0.238	0.131
	TAcells	0.469	-0.038	0.952	0.281	0.129
	WNT2B+	-0.402	-0.772	0.043	0.245	0.126
	WNT5B+	-0.458	-0.935	0.067	0.296	0.129
	Tcells	-0.438	-0.778	0.043	0.263	0.117
	Bcells	-0.601	-1.047	-0.163	0.229	0.126
	Monocytes	-0.421	-0.817	0.044	0.258	0.124
LP - H vs. I	Microvascular	-0.612	-1.188	0.040	0.352	0.126
	Glia	-0.935	-1.558	-0.240	0.341	0.126
	InflammatoryFibroblasts	0.397	-0.155	1.425	0.481	0.126
	WNT2B+Fos-lo1	-0.403	-1.030	0.097	0.332	0.126
	WNT2B+Fos-hi	-0.160	-0.838	0.165	0.292	0.126
	ILCs	-0.261	-0.820	0.104	0.272	0.126
	NKs	-0.491	-0.964	0.025	0.277	0.126
	MT-hi	-0.186	-0.813	0.170	0.280	0.126
	CD4+ActivatedFos-hi	-0.830	-1.333	-0.310	0.272	0.126
	CD4+ActivatedFos-lo	-1.167	-1.686	-0.654	0.276	0.126
	CD8+IL17+	-0.127	-0.685	0.129	0.237	0.126
	CD8+LP	-0.732	-1.044	-0.409	0.168	0.126
	Plasma	-0.925	-1.217	-0.580	0.174	0.126
	Macrophages	-0.259	-0.811	0.097	0.266	0.126
	CD4+T	-0.331	-0.693	0.027	0.213	0.118
	WNT2B+	-0.683	-1.360	0.073	0.453	0.122
	WNT5B+	-0.803	-1.551	0.051	0.489	0.125
	Bcells	-0.125	-0.474	0.089	0.162	0.122
	Monocytes	-0.365	-0.848	0.068	0.283	0.120
	Fibroblasts	-0.136	-1.052	0.125	0.362	0.116

Table S3. Credible effects, highest density intervals, standard deviations and credibility threshold δ determined by tascCODA on biopsies from Smillie et al. (2019), $\phi = 0$. Abbreviations for scenarios: Healthy (H), Non-inflamed (N), and Inflamed (I). Credible effects were only found for one of six scenarios.

Scenario	Node	Effect	HDI 3%	HDI 97%	SD	δ
Epithelium - H vs. I	CyclingTA	-0.394	-0.669	0.010	0.193	0.074
	TA1	0.151	-0.023	0.496	0.176	0.074
	Immaturecells	-0.117	-0.500	0.026	0.177	0.074
	Absorptive	-0.553	-0.853	-0.205	0.179	0.074
	Immune	0.149	-0.015	0.324	0.108	0.074
LP - H vs. N	Plasma	-0.086	-0.524	0.037	0.185	0.066
	Tcells	-0.612	-0.796	-0.425	0.100	0.066
	Bcells	-0.761	-1.011	-0.380	0.173	0.066
	Monocytes	-0.315	-0.618	0.024	0.216	0.066
	Myeloid	-0.113	-0.511	0.035	0.184	0.066
	Epithelial	0.145	-0.013	0.322	0.106	0.066
	Stromal	-0.303	-0.483	0.007	0.143	0.066
LP - H vs. I	CD4+ActivatedFos-lo	-0.463	-0.967	0.034	0.316	0.063
	Plasma	-0.747	-0.963	-0.528	0.117	0.063
	CD4+T	-0.425	-0.708	-0.055	0.164	0.063
	Monocytes	-0.269	-0.568	0.019	0.197	0.063
	Fibroblasts	-0.154	-0.638	0.038	0.222	0.063
	Stromal	-0.525	-0.835	-0.148	0.184	0.063

Table S4. Credible effects found by tascCODA comparing the gut microbiome of healthy controls and IBS patients from Labus et al. (2017) for varying aggregation levels ϕ.

ϕ	Kingdom	Phylum	Class	Order	Family	Genus	Effect
-5	Bacteria	Firmicutes					-0.313
0	Bacteria	Bacteroidota	Bacteroidia	Bacteroidales	Tannerellaceae	Parabacteroides	-0.156
0	Bacteria	Bacteroidota	Bacteroidia	Bacteroidales	Bacteroidaceae	Bacteroides	-0.662
0	Bacteria	Firmicutes	Clostridia	Oscillospirales			-0.232
5	Bacteria	Bacteroidota	Bacteroidia	Bacteroidales	Tannerellaceae	Parabacteroides	-0.845
5	Bacteria	Bacteroidota	Bacteroidia	Bacteroidales	Bacteroidaceae	Bacteroides	-1.001
5	Bacteria	Bacteroidota	Bacteroidia	Bacteroidales	Prevotellaceae	Prevotella	-0.413
5	Bacteria	Firmicutes	Clostridia	Lachnospirales	Lachnospiraceae	Agathobacter	-0.610
5	Bacteria	Firmicutes	Clostridia	Oscillospirales	Ruminococcaceae	Subdoligranulum	-0.224
5	Bacteria	Firmicutes	Clostridia	Oscillospirales	Ruminococcaceae	Faecalibacterium	-0.252
5	Bacteria	Firmicutes	Negativicutes	Acidaminococcales	Acidaminococcaceae	Phascolarctobacterium	-0.250
5	Bacteria	Firmicutes	Clostridia	Oscillospirales	Ruminococcaceae		-0.340

Table S5. Credible effects found by tascCODA ($\phi = 5$) comparing the gut microbiome of four different subtypes of IBS to all other samples. Original data by Labus et al. (2017).

Subtype	Kingdom	Phylum	Class	Order	Family	Genus	Effect
IBS-C	Bacteria	Bacteroidota	Bacteroidia	Bacteroidales	Bacteroidaceae	Bacteroides	-0.426
IBS-C	Bacteria	Firmicutes	Clostridia	Lachnospirales	Lachnospiraceae	Anaerostipes	0.438
IBS-C	Bacteria	Firmicutes	Clostridia	Lachnospirales	Lachnospiraceae	Agathobacter	-0.819
IBS-C	Bacteria	Firmicutes	Clostridia	Oscillospirales	Ruminococcaceae	Ruminococcus	-0.262
IBS-C	Bacteria	Firmicutes	Clostridia	Oscillospirales	Ruminococcaceae	Faecalibacterium	-0.320
IBS-D	Bacteria	Bacteroidota	Bacteroidia	Bacteroidales	Tannerellaceae	Parabacteroides	-0.392
IBS-D	Bacteria	Bacteroidota	Bacteroidia	Bacteroidales	Bacteroidaceae	Bacteroides	-1.405
IBS-M	Bacteria	Bacteroidota	Bacteroidia	Bacteroidales	Tannerellaceae	Parabacteroides	-0.424
IBS-M	Bacteria	Firmicutes	Clostridia	Lachnospirales	Lachnospiraceae	Blautia	0.799
IBS-M	Bacteria	Firmicutes	Clostridia	Oscillospirales	Ruminococcaceae	Faecalibacterium	-0.285
IBS-unspecified	Bacteria	Firmicutes	Clostridia	Peptostreptococcales-Tissierellales	Peptostreptococcaceae	Romboutsia	0.259

REFERENCES

Bai, R., Rockova, V., and George, E. I. (2020). Spike-and-Slab meets LASSO: A review of the Spike-and-Slab LASSO

Büttner, M., Ostner, J., Müller, C. L., Theis, F. J., and Schubert, B. (2020). scCODA: A bayesian model for compositional single-cell data analysis. doi:10.1101/2020.12.14.422688

Callahan, B. J., McMurdie, P. J., Rosen, M. J., Han, A. W., Johnson, A. J. A., and Holmes, S. P. (2016). DADA2: High-resolution sample inference from illumina amplicon data. *Nat. Methods* 13, 581–583. doi:10.1038/nmeth.3869

Labus, J. S., Hollister, E. B., Jacobs, J., Kirbach, K., Oezguen, N., Gupta, A., et al. (2017). Differences in gut microbial composition correlate with regional brain volumes in irritable bowel syndrome. *Microbiome* 5, 49. doi:10.1186/s40168-017-0260-z

Quast, C., Pruesse, E., Yilmaz, P., Gerken, J., Schweer, T., Yarza, P., et al. (2013). The SILVA ribosomal RNA gene database project: improved data processing and web-based tools. *Nucleic Acids Res.* 41, D590–6. doi:10.1093/nar/gks1219

Ročková, V. and George, E. I. (2018). The Spike-and-Slab LASSO. *J. Am. Stat. Assoc.* 113, 431–444. doi:10.1080/01621459.2016.1260469

Smillie, C. S., Biton, M., Ordovas-Montanes, J., Sullivan, K. M., Burgin, G., Graham, D. B., et al. (2019). Intra- and inter-cellular rewiring of the human colon during ulcerative colitis. *Cell* 178, 714–730.e22. doi:10.1016/j.cell.2019.06.029

Tibshirani, R. (1996). Regression shrinkage and selection via the lasso. *J. R. Stat. Soc. Series B Stat. Methodol.* 58, 267–288

Yilmaz, P., Parfrey, L. W., Yarza, P., Gerken, J., Pruesse, E., Quast, C., et al. (2014). The SILVA and “all-species living tree project (LTP)” taxonomic frameworks. *Nucleic Acids Res.* 42, D643–8. doi:10.1093/nar/gkt1209
